# Mucinous carcinoma originating in the peritoneum diagnosed by an ascites cell block: a case report

**DOI:** 10.1186/s13256-020-02435-4

**Published:** 2020-07-14

**Authors:** Sachiko Nagao, Motoki Matsuura, Masato Tamate, Shintaro Sugita, Tsuyoshi Saito

**Affiliations:** 1 Department of Obstetrics and Gynecology, Sapporo Medical University, South 1 West 16, Chuo-ku, Sapporo, 060-8543 Japan; 2 Department of Obstetrics and Gynecology, Kushiro City General Hospital, Kushiro, Hokkaido Japan; 3 Department of Surgical Pathology, Sapporo Medical University, Sapporo, Japan

**Keywords:** Peritoneal carcinoma, Mucinous carcinoma, Cell block method

## Abstract

**Background:**

Peritoneal carcinoma is a rare disease that is diagnosed and treated in a manner similar to ovarian cancer. In most cases, the histological type is serous carcinoma, and chemotherapy is effective. However, there are a few case reports of mucinous peritoneal carcinoma. We inferred the histological type before surgery using an ascites cell block sample, which was useful for determining the treatment plan.

**Case presentation:**

Our patient was a 60-year-old Japanese woman. She presented with a feeling of fullness in the abdomen. A computed tomographic scan showed a large quantity of ascitic fluid and thickening of the greater omentum, as well as thickening of the peritoneum at the pouch of Douglas and diaphragm. Hence, peritoneal carcinoma was suspected. The tumor markers carcinoembryonic antigen, cancer antigen 19-9, and cancer antigen 125 were all increased, and no malignant findings were observed in the uterus or ovaries. Cells suggestive of carcinoma were found in the ascitic fluid, and immunostaining by the cell block method suggested the possibility of mucinous carcinoma. The preoperative chemotherapy strategy was changed to short courses, and tumor reduction surgery was planned. Similar to the suspicion before surgery, the pathology results indicated mucinous carcinoma, and the therapeutic effect of chemotherapy was grade 0.

**Conclusions:**

Determining whether peritoneal carcinoma is serous carcinoma is important for therapy and prognostic prediction. In this case, we encountered a patient for whom surgery was chosen because of drug therapy resistance inferred through histological type estimation using the cell block method. Inferring the histological type by cell block preparation is useful for diagnosis and treatment selection.

## Background

Peritoneal carcinoma is a rare disease that is diagnosed and treated in a manner similar to ovarian cancer. Prognostic factors include the histological type, degree of the residual lesion after surgery, and presence of lymph node metastases. The most common histological type is serous carcinoma, and chemotherapy is usually effective against this type, but the other histological types are usually resistant to treatment [1, 2]. We present a case of a patient with peritoneal carcinoma whom we diagnosed with mucinous carcinoma by the cell block method, which was critical in determining the optimal treatment plan.

## Case presentation

Our patient was a 60-year-old Japanese woman. She had no relevant medical, family, or psychosocial history or past interventions. She had visited our department 3 months prior with a primary complaint of abdominal fullness and loss of appetite. She had not been to an obstetrician/gynecologist since a cancer examination 12 years prior; the result of uterine cervical cytology was negative. Her abdominal circumference was 85 cm. She had been pregnant and delivered twice. Her mother had breast cancer.

Transvaginal ultrasound and a contrast-enhanced computed tomographic (CT) scan showed a large quantity of ascitic fluid and thickening of the greater omentum, as well as thickening of the peritoneum at the pouch of Douglas, surface of the liver, and diaphragm; peritoneal carcinoma was suspected. A hematological test at the initial visit did not show anemia or increased leukocytes. However, all of the patient’s tumor markers were elevated (carcinoembryonic antigen, 14.8 ng/ml; cancer antigen 125, 39.8 U/ml; and cancer antigen 19-9, 500.9 U/ml), and her lactate dehydrogenase, C-reactive protein, and D-dimer levels were also increased, which were believed to be effects of the tumor. Images did not show overt abnormal findings, such as masses in the uterus or uterine appendages on either side, and the result of uterine cervix/body cytology was negative. Cytology by paracentesis showed findings suggestive of adenocarcinoma, and the treatment plan was two or three courses of preoperative chemotherapy followed by debulking surgery.

**Fig. 1:**
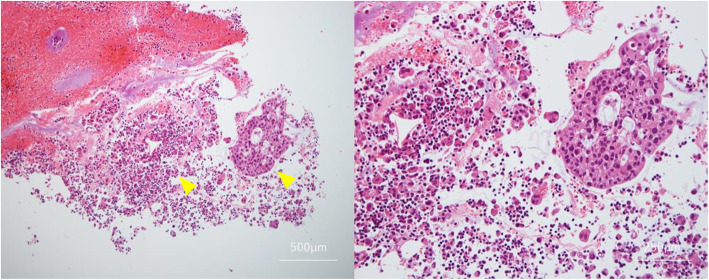
Ascites cytology. Mucus-producing structures suggestive of mucinous carcinoma were observed (arrowheads: tumour cells with alveolar, luminal, and cribriform structures)

Cytology results showed tumor cells with alveolar, luminal, and cribriform structures (Fig. 1). Given that the tumor markers were not as elevated as the severe imaging findings and clinical symptoms might have suggested, a hematological type other than serous carcinoma was considered, and an ascites cell block sample was prepared. The results of immunostaining with the cell block sample showed that cytokeratin 7 (CK7) and CK20 stained positive for periodic acid–Schiff (PAS), and a mucus-producing tumor was suspected. It was possible that the site of the primary tumor was in an area other than the ovaries, such as the pancreas or bile ducts. To render an exclusive diagnosis, upper and lower gastrointestinal endoscopy and a three-phase CT scan of the liver, gallbladder, and pancreas were performed; however, no obvious lesions were found. Tentatively, we diagnosed the patient with stage IIIC mucinous peritoneal carcinoma.

**Fig. 2:**
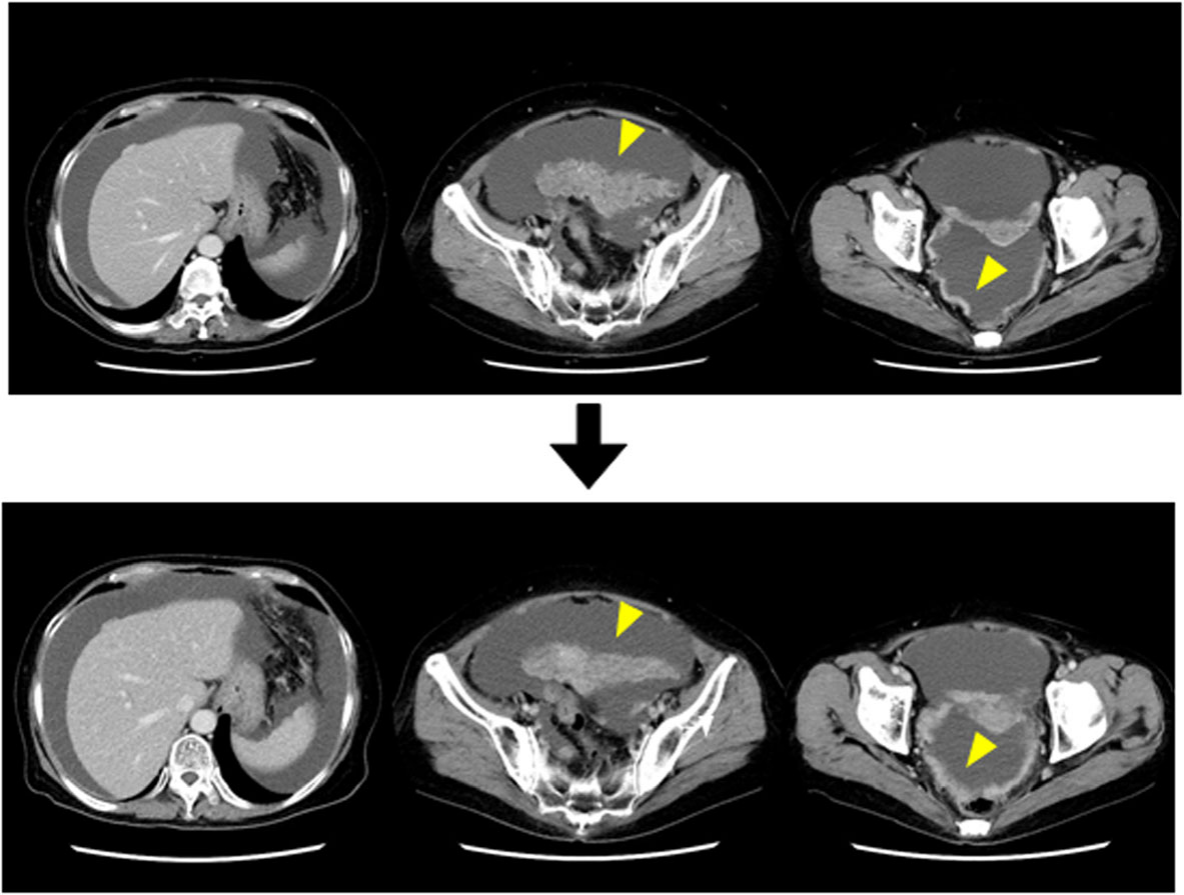
Preoperative contrast-enhanced computed tomography. Two courses of preoperative chemotherapy were administered, but the ascites and lesions in the greater omentum and peritoneum remained unchanged (arrowheads)

Clinically, the treatment plan for mucinous carcinoma is no different from that for stage IIIC peritoneal carcinoma. However, because mucinous carcinoma is likely to be more resistant to treatment than serous carcinoma, the originally planned three courses of preoperative chemotherapy consisting of docetaxel + carboplatin (CBDCA) (hereinafter referred to as “DC therapy”) was changed to two courses, and surgery was planned. The observed adverse events of chemotherapy included grade 3 neutropenia, grade 2 anemia, and grade 3 anorexia and hypoalbuminemia (occurring with abdominal fullness due to ascitic fluid retention). Paracentesis was performed twice during hospitalization for the first course of chemotherapy, removing 2500 ml of fluid each time, and concentrated ascites reinfusion therapy was performed with the second paracentesis. A CT scan following preoperative chemotherapy did not show an increase in ascitic fluid, and because no major changes had occurred in the peritoneal lesions, the disease state was considered stable (Fig. 2).

In discussing surgery with the patient, the possibility of having to perform enterectomy and artificial anus creation as a result of removing the peritoneal and greater omentum lesions was explained. Subsequently, joint surgery was performed at our institution’s Department of Surgery at 3 months after the initial consultation. No visually abnormal intrapelvic findings were observed in the uterus or bilateral uterine appendages, though diffuse peritoneal lesions were observed on the vesicouterine pouch peritoneum, pouch of Douglas, and surface of the sigmoid colon. The ascites was serous, and no overt lesions were observed on the appendix. Our department extracted the uterus and bilateral uterine appendages and removed the peritoneum on the side of the bladder in the pelvic cavity, at which point we requested lower anterior resection from our institution’s Department of Surgery. The greater omentum exhibited hard thickening throughout, and resection was possible because the distance from the stomach had been maintained. However, the area near the left and right colic flexures had moved close to the intestines; therefore, only partial extraction of the greater omentum was performed, leaving behind part of the tumor. The residual lesion was less than 1 cm; the operation time was 3 hours and 30 minutes; the bleeding volume was 3310 ml; and 10 units of erythrocytes and 10 units of fresh frozen plasma were transfused. The uterine appendages were 110 g; the omentum was 400 g; the sigmoid colon was 220 g; and the ascites volume was 1500 ml.

**Fig. 3:**
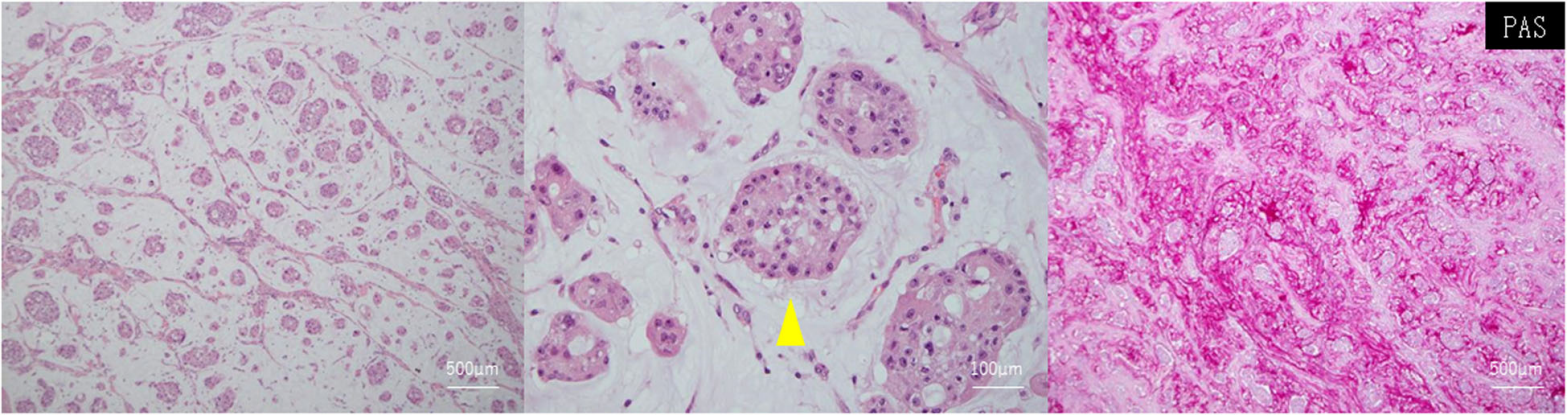
Pathological findings. Tumor cells with oval nuclei and clear nucleoli were proliferating in an alveolar form and a luminal form in a mucus lake (arrowhead) that stained positive for periodic acid–Schiff and Alcian blue. These findings were consistent with mucinous carcinoma

The pathology results showed tumor cells with oval nuclei and clear nucleoli proliferating in an alveolar form and a luminal form in a mucus lake that stained positive for PAS and Alcian blue; some of the tumor cells produced mucus. Therefore, the diagnosis was mucinous carcinoma, which corresponded to the suspicion based on the preoperative cell block sample (Fig. 3). Tumors were observed on the serous membrane side of the uterus and uterine appendages, the serous side of the sigmoid colon, and the peritoneal nodules, whereas no tumors were observed on either ovary, the endometrium, the cervix of the uterus, or the mucosa of the sigmoid colon. Hence, the primary tumor was believed to have originated from the peritoneum. Immunostaining results were positive for CK7 and CK20 and negative for CDX-2 (caudal type homeobox 2), TTF-1(thyroid transcription factor-1), p53, D2-40 (podoplanin), and ER (estrogen receptor), and no K-ras abnormalities were observed. The therapeutic effect of chemotherapy was grade 0, and the diagnosis was treatment-resistant mucinous carcinoma.

Because of treatment resistance, the postoperative chemotherapy regimen was adjusted to gemcitabine + CBDCA + bevacizumab therapy (hereinafter referred to as “GC + BV therapy”). During the nine courses of postoperative chemotherapy, the tumors did not grow. However, when the switch to bevacizumab monotherapy was made, the peritoneal dissemination lesions grew. The chemotherapy plan was changed but was unable to suppress the disease state, and the patient died of the original disease because of peritonitis due to intestinal perforation by the peritoneal dissemination lesions at 1 year and 9 months after the initial operation was performed.

## Discussion and conclusions

Peritoneal carcinoma develops from the primitive mesothelium and occurs in the peritoneum and in multiple locations, from the peritoneal mesothelium to the ovarian surface epithelium. Its pathology is similar to that of ovarian carcinoma, and it is usually serous carcinoma. Peritoneal carcinoma very rarely occurs as clear cell carcinoma, mucinous carcinoma, or endometroid carcinoma [1, 2]. Currently, the diagnostic criteria of the Gynecologic Oncology Group are widely used [3].

Because the histopathological findings and success rate of chemotherapy are similar to those of ovarian cancer and the number of cases is limited, peritoneal carcinoma is often treated together with ovarian carcinoma and fallopian tube carcinoma as a Mullerian duct–derived carcinoma. However, strictly speaking, peritoneal cancer is of polyclonal origin, whereas ovarian serous carcinoma is of monoclonal origin [4]. Moreover, peritoneal carcinoma has racial characteristics [5], is strongly correlated with the *BRCA* gene, and frequently occurs after oophorectomy. Therefore, the question whether it should be treated in the same manner as ovarian cancer is controversial.

Risk factors for peritoneal carcinoma include age, fertility, and obesity. Subjective symptoms rarely occur in the initial stages. Peritoneal carcinoma manifests as a sense of abdominal fullness or a large quantity of ascitic fluid, and positive ascites cytology is usually observed [6]. The carcinoma tissue observed in ascites is also observed in endometrial cytology, accompanied by microscopic metastases to the serous membrane in approximately 50% of cases and visible metastases in approximately 30% of cases. At the initial visit, our patient’s cancer was already at an advanced stage, with a large quantity of ascitic fluid retention, metastases to the omentum, and positive ascites cytology.

Generally, the treatment strategy is based on advanced epithelial ovarian carcinoma and is intensive, with an initial tumor reduction operation followed by postoperative chemotherapy. The standard chemotherapies are docetaxel and cyclophosphamide (TC) therapy (paclitaxel + CBDCA), DC therapy, and dose-dense TC therapy. If the maximum diameter of the residual tumor is less than 1 cm, the prognosis is good; if the residual tumor can be reduced to the microscopic level, the prognosis is extremely good. In actual clinical practice, typically, a large quantity of ascites and pleural effusion is present at the initial examination, as described previously, which often deteriorates the patient’s general condition. Our patient also had a large quantity of ascites, a state that impaired her ability to consume food. In such cases, a minimally invasive operation, such as laparoscopy or exploratory laparotomy, is performed for diagnosis. Alternatively, the patient may be diagnosed with suspected peritoneal carcinoma or clinical peritoneal carcinoma, and preoperative chemotherapy may be performed first, with the aim of controlling the ascites. In our patient’s case, we selected the latter option and planned three courses of preoperative DC chemotherapy.

Because the effect of preoperative chemotherapy is believed to be greatly involved in the success of the initial tumor reduction operation and the prognosis of the disease, we performed immunohistological testing using the cell block method to diagnose the histological type using an ascites sample. The cell block method involves preparing a thin-sliced serial section by solidifying the obtained cell cytology sample and then fixating it with formalin and embedding it in paraffin. Similar to a histological diagnosis, immunostaining is a potential component of the cell block method. Its advantages include the ease of handling the samples as a paraffin block and that multiple samples of the same cell can be prepared with serial sections, semipermanent storage is possible, and the sections can be used for deoxyribonucleic acid (DNA) extraction.

In our patient’s case, the results of immunostaining were positive for both CK7 and CK20, suggesting the possibility of ovarian mucinous carcinoma, urinary tract epithelial tumor, pancreatic carcinoma, or cholangiocarcinoma. However, because the imaging tests did not show overt neoplastic lesions on these organs, peritoneal carcinoma was more actively suspected. The results of other immunostaining tests were negative for CDX-2, which is expressed in intestinal cells, and TTF-1, which is expressed in pulmonary carcinoma or thyroid carcinoma, and positive for PAS. Therefore, the possibility that the tumor was mucus-producing was believed to be high. As described above, chemotherapy can be expected to have therapeutic efficacy in serous carcinoma, which constitutes most cases of peritoneal carcinoma. However, other histological types of peritoneal carcinoma are likely to be resistant to treatment. It was believed that our patient had mucinous peritoneal carcinoma and that we should prioritize tumor reduction surgery over the expectation that three courses of preoperative chemotherapy would be effective. Indeed, a contrast CT scan after two courses of chemotherapy did not show significant tumor reduction or a decrease in the ascites; thus, the disease was stable. Therefore, we changed the treatment plan from three courses to two courses of chemotherapy, followed by early surgery. The therapeutic effect on the pathology specimen was indeed grade 0.

For the postoperative chemotherapy regimen, the cancer was expected to be resistant to platinum drugs because preoperative DC therapy had shown a poor therapeutic effect. When considering bevacizumab combination therapy to control the ascites, and we selected GC + BV therapy, which was shown to be effective against recurrent ovarian cancer in a phase III study (OCEANS study [Ovarian Cancer Study Comparing Efficacy and Safety of Chemotherapy and Anti-Angiogenic Therapy in Platinum-Sensitive Recurrent Disease]) [7].

The conversion to a treatment plan for our patient that included tumor reduction surgery at an early stage demonstrates the efficacy of the cell block method. The possibility exists that we should have more thoroughly considered whether extraction of the recurrent tumor might have improved the prognosis.

In conclusion, the ability to classify peritoneal carcinoma as serous carcinoma is critical for maximizing the therapeutic effect and prognostic capability. In our patient’s case, suspicion of the histological type using the cell block method was useful in determining the optimal treatment strategy.

## Data Availability

Not applicable.
